# Impact of risk factors, early rehabilitation and management of lymphedema associated with breast cancer: a retrospective study of breast Cancer survivors over 5 years

**DOI:** 10.1186/s12905-024-03062-7

**Published:** 2024-04-06

**Authors:** Slobodan Tomić, Goran Malenković, Ermina Mujičić, Armin Šljivo, Sanja D. Tomić

**Affiliations:** 1 Faculty of Medicine of University of Novi Sad, Novi Sad, Serbia; 2 Department of Nursing, Faculty of Medicine, University of Novi Sad, Novi Sad, Serbia; 3 Clinical Center of University of Sarajevo, Sarajevo, Bosnia and Herzegovina

**Keywords:** Secondary lymphedema, Breast cancer, Surgery, Occurrence, Severity

## Abstract

**Background:**

Breast cancer-related lymphedema (BCRL) is a potentially disabling and often irreversible consequence of breast cancer treatment, caused by the mechanical incompetence of the lymphatic system, resulting in reduced drainage capacity and functional overload due to an excessive volume of interstitial fluid surpassing the system’s transport capacity in the arm. We wanted to determine the impact and explore the differences in independent risk factors for the occurrence of BCRL; incidence of BCRL over a five-year period at the Institute of Oncology Vojvodina in Sremska Kamenica and to answer the research question regarding the influence of the prehabilitation program on the overall incidence of BCRL during the observed five-year period.

**Methods:**

From 2014 to 2018, a retrospective study was conducted at the Institute of Oncology of Vojvodina in Sremska Kamenica, analyzing female patients who had undergone breast cancer surgery.

**Results:**

The study included 150 breast cancer patients who developed secondary lymphedema following surgery with the mean age of 59.2 ± 11.3 years. Fluctuations in hospitalization rates were observed over the five-year period, with the highest number of admissions in 2014 (24.0%) and a decline in 2018 (14.0%). The most common surgical procedure performed was left quadrantectomy (24.0%), followed by right quadrantectomy (20.0%) and left amputation (15.3%). The mean number of removed lymph nodes was 15.2 ± 6.1, with no statistically significant association between the number of removed lymph nodes and the manifestation of secondary lymphedema. The severity of secondary lymphedema varied based on patient age, with a higher incidence of moderate and severe lymphedema observed in patients aged 61 years and older. Patients who underwent radical surgery were more likely to experience severe lymphedema compared to those who had conservative surgery, although this difference was not statistically significant.

**Conclusion:**

In our study, the type of surgery, elapsed time since surgery, and the number of removed lymph nodes were not influencing factors for the occurrence of BCRL. However, concerning its severity, a greater number of systemic therapy modalities combined with radiotherapy were associated with a more frequent occurrence of mild and moderate BCRL. Also, the severity of BCRL varied among different age groups, with a higher incidence of moderate and severe lymphedema observed in patients aged 61 years and older. Ultimately, improving the quality of life for individuals affected by secondary lymphedema remains a crucial goal in the field of oncology.

## Background

Lymphedema associated with breast cancer (BCRL) is a potentially disabling and often irreversible complication of breast cancer treatment. It is believed to occur as a result of reduced drainage capacity or functional overload of the lymphatic system when the volume of interstitial fluid exceeds the existing transport capacity of the lymphatic system in the arm, due to the mechanical incapacity of the lymphatic system [1–3]. Studies have elucidated the complex interaction of inflammatory processes, disturbed lymphatic remodeling, and impaired lymphatic angiogenesis in the intricate pathogenesis of BCRL, involving multiple risk factors [4–6]. The likelihood of developing BCRL largely depends on risk factors that can be divided into two categories. One group consists of patient-specific or dependent factors, while the other group includes treatment-specific independent risk factors for breast cancer. Dependent factors include body mass index (BMI) at the time of diagnosis, subclinical edema, and cellulitis on the treated side [7]. Independent risk factors for BCRL related to treatment include the type of surgical intervention, the number of removed lymph nodes, radiation to regional lymph nodes, and chemotherapy [8–12]. Regarding the timeframe, BCRL can manifest at different time intervals during the postoperative period, with the majority of cases occurring within the first 2 years after surgery [3]. However, it is important to note that BCRL can develop even several years after the initial treatment, emphasizing the need for long-term monitoring and support for breast cancer survivors [4]. Therefore, early detection and timely interventions are integral parts of BCRL prevention. Diagnosing preclinical lymphedema can be challenging and requires preoperative assessment and monitoring [13–17]. The diagnosis of BCRL is based on a detailed consideration of risk factors, associated symptoms, clinical signs, and physical examination. Literature provides a wide range of non-invasive methods for assessing secondary lymphedema of the arm, including sequential measurements of arm circumference, volumetric measurements, and tissue tonometry [7, 18]. Differences of 2 cm or more in arm circumference recorded at least at one measurement site, or when translated into volume, an increase of 10% or an increase of 200 ml at any time compared to the opposite arm, constitute clinical indicators for the diagnosis of BCRL [4–6]. The four stages of lymphedema based on the International Society of Lymphology framework are described as follows: Stage 0 - subclinical with no visible changes; Stage 1 - soft edema, without dermal fibrosis and with pitting that resolves upon elevation of the limb; Stage 2 - moderate with decreased elasticity due to evolving dermal fibrosis and no reduction in swelling with limb elevation; and Stage 3 - chronic and irreversible [19]. Based on the severity, breast cancer-related lymphedema can be classified into three categories: mild, moderate, and severe. The lack of standardized diagnostic criteria and consistent diagnostic methods for BCRL results in varying reported incidence rates ranging from less than 5% to over 50% [3–5].

Taking all of the above into consideration, with this study, we aimed to address several questions. Firstly, we wanted to determine the impact and explore differences in independent risk factors for the development of BCRL. Secondly, our goal was to establish the incidence of BCRL over a five-year period at the Institute of Oncology of Vojvodina in Sremska Kamenica. Our ultimate aim was to answer the research question regarding the influence of prehabilitation on the overall incidence of BCRL during the observed five-year period.

## Methods

### Patients and study design

A retrospective study was conducted at the Institute of Oncology of Vojvodina (IOV) in Sremska Kamenica on a sample of patients who underwent breast cancer surgery from 2014 to 2018. The patient sample was formed based on the following criteria: surgical treatment of breast cancer with either breast-conserving or radical surgery with axillary dissection, application of systemic therapy (chemotherapy, hormonal therapy, biological therapy, individually or in combination), undergoing radiation therapy, and participation in an early rehabilitation program. Patients who underwent sentinel node biopsy and those with oncoplastic surgical approaches were not included in the study.

### Ethical approval and informed consent

The study was approved by the Ethics Committee of the Institute of Oncology of Vojvodina in Sremska Kamenica and the Ethics Committee of the Faculty of Medicine in Novi Sad (01–39/63). Every individual involved in the study was thoroughly briefed on the nature of the data employed, and they willingly and knowingly gave their informed consent for its use in the research.

### Data collection and study adjustment

Relevant patient data collected from medical histories for the study included: age, time elapsed since BCRL, type of surgery, number of removed lymph nodes, values of measured sequential circumferences of limbs, severity of lymphedema, and types of therapeutic modalities (chemotherapy, biological therapy, hormonal therapy, and radiotherapy).

The criterion for diagnosing BCRL was the presence of a circumference difference of 2 cm at least at one of the 5 measurement levels compared to the contralateral arm. The assessment of upper limb circumferences was performed at five symmetric levels: over the metacarpophalangeal joints; over the wrist joint; 10 cm below the olecranon; over the olecranon; and 10 cm above the olecranon. The lymphedema circumference, expressed as the ratio between the unaffected arm’s circumference and the affected arm’s circumference, was calculated using the following formula: [(total circumference of unaffected arm - total circumference of affected arm) / total circumference of healthy arm] × 100. Based on severity, BCRL was classified into three categories: mild lymphedema (minimal) - an increase in circumference from 2 to 2.9 cm; moderate lymphedema - an increase in circumference from 3 to 4.9 cm; and severe lymphedema - an increase in circumference of 5 cm or more.

All patients included in the study, who underwent surgery at the Institute of Oncology of Vojvodina in Sremska Kamenica, were enrolled in the early rehabilitation program. This program consists of active and/or actively assisted exercises aimed at maintaining and increasing mobility and muscle strength of the operated region, as well as the entire body, towards postural and functional reeducation. The early rehabilitation program promotes a set of six to seven exercises performed in standing, lying, and sitting positions up to the pain threshold and without fatigue, 2–3 times daily, gradually increasing the number of repetitions.

As rehabilitation begins on the second postoperative day, the difficulties that patients may face during this period can serve as potential barriers to the implementation of the exercise program. These difficulties include concerns about the illness, a subjective sense of shoulder joint stiffness, pain, lack of motivation, presence of fatigue and weakness, lack of interest, fear of injury, and fear related to the presence of the drainage system. For these reasons, exercises are conducted under the supervision of a physiotherapist to create an individual plan for each patient.

The main components of the individually tailored exercise plan include the intensity, frequency, and duration of physical activity, the selection of exercises to be applied both during hospitalization and at home until the first follow-up, as well as their sequence, duration, and number of repetitions. Each session begins with deep breathing and light warm-up exercises, gradually increasing the number of repetitions for each exercise. General recommendations advise performing selected five to six exercises daily during hospitalization, initially one to two times a day under the supervision of a physiotherapist. The number of repetitions for each exercise starts with two to three times (maximum five) initially, and after the removal of the drainage and in home conditions, the number of repetitions gradually increases but does not exceed a maximum of 10 repeated movements for each exercise. After discharge, follow-up visits are scheduled for 3 weeks, then for 1 month, and further as needed. The effectiveness of the early rehabilitation program has been documented in previous studies [20, 21].

### Statistical analysis

The data collected during the research were checked for validity, encoded, and entered into a specially created database on a personal computer. After data entry, their statistical analysis and processing were carried out using the IBM SPSS (Statistical Package for Social Sciences) version 26. Descriptive and inferential statistical methods were employed in the statistical analysis and data processing. Numeric variables were presented through mean values (arithmetic mean) and measures of variability (range, standard deviation), while categorical variables were presented through frequencies and percentages.

Testing differences in the frequency distribution for nominal variables was performed using the Pearson χ2 test with Yates’ correction for continuity, and for ordinal variables, the Mantel-Haenszel χ2 test was applied. In all analyses, *p* ≤ 0.05 was considered statistically significant, and the results were presented graphically and in tabular form, with the graphs created using the Excel 2016 software package.

## Results

**Fig. 1:**
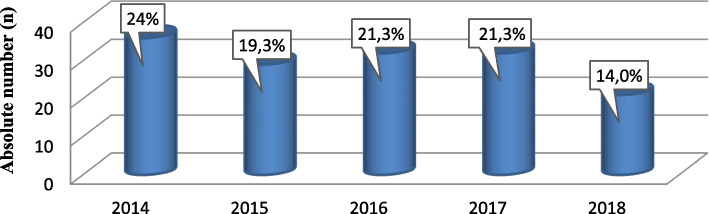
*Incidence of BCRL, 2014–2018 years*

The study included a total of 150 patients, with an average age of 59.2 ± 11.3, where the youngest patient was 23 and the oldest was 82 years old. Out of the 150 patients diagnosed with BCRL, in 2014, the highest number was recorded, with 36 patients (24.0%), slightly fewer in 2015, with 29 patients (19.3%), while in 2016 and 2017, an equal number of patients, 32 each (21.3%), was observed. In 2018, there was a decline in the number of patients with BCRL, with 21 patients (14.0%) (Fig. 1).

Regarding surgical procedures, the largest proportion of patients underwent left quadrantectomy, with 36 (24.0%) patients, followed by right quadrantectomy with 30 (20.0%) patients. Left amputation was performed on 23 (15.3%) patients, while segmentectomy was conducted on 19 (12.7%) patients. Right mastectomy was carried out in 16 (10.7%) patients, and right amputation in 14 (9.3%) patients. Left mastectomy was performed in 11 (7.3%) patients, while tumor resection, or tumorectomy, was only performed in one patient, representing 0.7% of the total. The mean time elapsed since the surgery was 15.1 months, with the shortest period from the operation at the time of data analysis being 1 month, and the longest period being 80 months. The largest number of patients, 75 (54.91%), received a combination of chemotherapy and hormonal therapy, while only two patients (1.63%) had a combination of hormonal and biological therapy (Table 1).

**Table 1:** Distribution of patients according to clinical characteristics

Treatment modalities		
Surgical procedures	*N* = 150	
	*n*	%
Tumorectomy	1	0.7
Mastectomy left	11	7.3
Mastectomy right	16	10.7
Amputation left	23	15.3
Amputation right	14	9.3
Quadrantectomy left	36	24.0
Quadrantectomy right	30	20.0
Segmentectomy	19	12.7
**Systemic therapy**		
Chemotherapy	13	8.6
Hormonal therapy	25	16.6
Chemotherapy + hormones	75	50.0
Chemotherapy + biological	17	11.3
Hormonal + biological	2	1.3
Chemotherapy + hormonal + biological	18	12.0

The mean time elapsed since the surgery was 15.1 months, with the shortest period from the operation at the time of data analysis being 1 month, and the longest period being 80 months (Table 2).

**Table 2:** Average values and measures of variability of the time elapsed from each surgery to the onset of BCRL

Surgical procedures	n	Min	Max	M	SD
Segmentectomy	19	1.0	56.0	15.4	17.3
Right quadrantectomy	30	1.0	64.0	13.2	14.6
Left quadrantectomy	36	1.0	80.0	14.3	17.0
Right amputation	14	1.0	38.0	12.3	10.4
Left amputation	23	1.0	68.0	15.6	18.6
Right mastectomy	16	1.0	57.0	14.9	17.9
Left mastectomy	11	7.0	53.0	25.5	17.3
Tumorectomy	1	2.0	2.0	2.0	.

Among the patients, 103 (68.7%) were diagnosed with mild BCRL, 32 (21.3%) had moderate BCRL, and 14 (9.3%) experienced severe BCRL. When comparing the BCRL categories based on the type of surgery, specifically conservative or radical surgical intervention, it is evident that a greater proportion of patients who underwent conservative surgery had mild BCRL compared to those who had radical surgery (63.1% vs 36.9%). Furthermore, a higher number of patients who underwent radical surgery were diagnosed with severe BCRL, as opposed to those who had conservative surgery (64.3% vs 35.7%). However, the observed differences are not statistically significant (Pearson chi-squared test; χ^2 = 5.495; df = 2; *p* = 0.064) (Table 3). The severity of BCRL was also not statistically significant when examined in relation to the applied therapy (Pearson chi-squared test; χ^2 = 5.196; df = 6; *p* = 0.591). The distribution and difference in the severity level of BCRL based on the applied therapy are presented in Table 3. Patients who received multiple therapy modalities more frequently had mild and moderate BCRL compared to patients who received one or two therapy modalities (61.2 and 66.8% vs 38.8 and 31.3%). The manifestation of a severe form of BCRL in relation to the applied therapy was the same in both groups of patients (Table 3).

**Table 3:** Distribution and difference in the severity level of BCRL based on the type of surgery and the number of applied therapy

***BCRL severity***	Type of surgery					
	Conservative surgery		Radical surgery		Total	
	n	%	n	%	n	%
Mild	65	63.1	38	36.9	103	100.0
Moderate	15	46.9	17	53.1	32	100.0
Severe	5	35.7	9	64.3	14	100.0
*Pearson chi-squared test; χ*^2^*= 5495; df = 2; p = 0,064*						
***BCRL severity***	Systemic therapy and radiotherapy					
	Radiotherapy and one systemic therapy		Radiotherapy and two or three systemic therapies		Total	
	n	%	n	%	n	%
Mild	40	38.8	63	61.2	103	100.0
Moderate	10	31.3	22	66.8	14	100.0
Severe	7	50.0	7	50.0	14	100.0
*Pearson chi-squared test; χ*^2^*= 1497; df = 2; p = 0,473*						

Regarding the number of removed lymph nodes, no statistically significant differences were observed in the manifestation of BCRL (Mantel-Haenszel chi-squared test; χ^2^ = 3.150; df = 1; *p* = 0.076) (Table 4). However, severe BCRL is more frequent in patients with a larger number of removed nodes compared to subjects with a smaller number of removed lymph nodes (78.6% vs. 21.4%). Comparing the categories of BCRL severity with the time elapsed since surgery, no statistically significant differences were observed (Mantel-Haenszel chi-squared test; χ^2^ = 0.287; df = 1; *p* = 0.592).

**Table 4:** *Distribution and difference in the severity level of BCRL based on the type of surgery* and the number of applied therapies

***BCRL severity***	Number of removed lymph nodes									
	≤ 12		13–16				≥ 17		Total	
	n	%	n		%		n	%	n	%
Mild	32	31.1	36		35.0		35	34.0	103	100.0
Moderate	7	21.9	14		43.8		11	34.4	32	100.0
Severe	3	21.4	0		0.0		11	78.6	14	100.0
Mantel-Haenszel chi-squared test; *χ*^2^ = 3.150; df = 1; p = 0.076										
***BCRL severity***	Elapsed time since surgery (months)									
	≤ 2		3–9		10–21		≥ 22		Total	
	n	%	n	%	n	%	n	%	n	%
Mild	27	26.2	27	26.2	25	24.3	24	23.3	103	100.0
Moderate	8	25.0	8	25.0	9	28.1	7	21.9	14	100.0
Severe	4	28.6	2	24.8	2	24.8	5	35.7	14	100.0
Mantel-Haenszel chi-squared test; *χ*^2^ = 0.287; df = 1; p = 0.592										

In comparing the severity categories of BCRL based on patient age, the highest number of patients with mild BCRL was found in the group aged ≤55 years (*n* = 39, 37.9%). Moderate BCRL was diagnosed in patients between the ages of 56 and 65 years, while severe BCRL was observed in half of the patients aged 66 years and older. Statistically significant differences were found when examining the severity of BCRL in relation to two age categories: patients ≤60 years old and those 61 years old and above (Mantel-Haenszel chi-squared test; χ^2 = 4.851; df = 1; *p* = 0.028). Specifically, a higher incidence of moderate and severe BCRL was observed among patients aged 61 years and older (Table 5.).

**Table 5:** Distribution and difference in the severity level of SLER in relation to the age of patients

*BCRL severity*	Age					
	≤ 60		≥ 61		Total	
	n	%	n	%	n	%
Mild	60	58.3	43	41.7	103	100.0
Moderate	15	46.9	17	53.1	32	100.0
Severe	4	28.6	10	71.4	14	100.0
*Mantel-Haenszel chi-squared test*; *χ*^2^ = 4.851; df = 1; p = 0.028						

## Discussion

This study is one of the first in the region to investigate the occurrence of BCRL after breast cancer surgery in Serbia and the West Balkan area. The study aimed to explore the correlation between various factors such as hospitalization rates, surgical procedures, number of removed lymph nodes, elapsed time since surgery, patient age, type of surgery, and applied therapy with the severity of BCRL. Our study population, which had different surgical approaches towards breast cancer removal, showed different hospitalization rates over a five-year period from 2014 to 2018. In our study, analyzing a sample of patients operated at the Institute of Oncology of Vojvodina in Sremska Kamenica during the period from 2014 to 2018, the highest frequency of BCRL was recorded in 2014. Out of a total of 232 breast cancer patients who underwent surgery, BCRL was diagnosed in 36 (15.51%) patients. The following year, 2015, had a higher number of patients, with 291 patients, but BCRL was diagnosed in 29 (9.96%) patients. The lowest frequency of BCRL was recorded in 2018, when out of 267 patients, BCRL was diagnosed in only 21 patients (7.86%). In 2016, BCRLwas diagnosed in 32 patients (11.8%) out of a total of 252 surgeries, while in 2017, BCRL was diagnosed in 32 patients (12.69%). A higher incidence of moderate and severe BCRL was observed among patients aged 61 years and older, while a larger proportion of patients who underwent conservative surgery experienced mild BCRL compared to those who had radical surgery. No differences were found when examining the categories of BCRL severity in relation to the time that had passed since the surgery and in relation to the applied therapy modalities. Regarding hospitalization rates, the data shows fluctuations over a five-year period. The composition of the study population, including patient demographics and disease stage, could have influenced the likelihood of hospitalization. Factors such as comorbidities, disease severity, and patient preferences might have varied over the years, leading to differences in hospitalization rates. Outpatient programs for managing lymphedema, which consist of physical therapy and surgical interventions, can significantly enhance the care and outcomes of patients who have multiple coexisting health conditions. Prevention of BCRL can be treatment-specific. Reducing the extent of axillary surgery in certain circumstances, mapping the lymphatic channels of the upper extremities during surgery, and reducing the amount of nodal radiation in appropriate cases can help in prevention. According to the data, the most frequently performed procedure among patients was left quadrantectomy, accounting for 24.0% of the cases, followed by right quadrantectomy, which represented 20.0% of the patients. Although the differences were not statistically significant, a higher proportion of patients who underwent conservative surgery had mild BCRL compared to those who had radical surgery. Conversely, severe BCRL was more frequently diagnosed in patients who underwent radical surgery. These findings indicate a potential association between the type of surgery and the severity of lymphedema, although further research is necessary to establish a conclusive correlation. However, in our study, there was no compelling evidence to define the number of lymph nodes removed in correlation with BCRL. Recent studies [22–24] have shed light on a potential positive correlation between the number of lymph nodes removed during surgical procedures and the likelihood of developing BCRL. In other words, these studies suggest that a higher number of lymph nodes removed may increase the risk of experiencing significant lymphedema symptoms as a secondary condition. Vicini et al. [25] showed a trend of increased lymphedema when four or more lymph nodes were removed; however, this was not statistically significant. Engel et al. [26] demonstrated that taking 10 or more lymph nodes was significantly associated with lymphedema. However, there is no consensus regarding the number and lymphedema. In a recent study of 936 patients, there was also no association between the number of nodes removed and lymphedema [27].

The findings indicated that there were no statistically significant differences in the severity of lymphedema based on the elapsed time since surgery. This suggests that the severity of secondary lymphedema may not be strongly influenced by the amount of time that has passed since the initial surgery. However, it should be noted that the average duration varied among different surgical procedures, suggesting potential differences in recovery or disease progression rates.

The older age group exhibited a higher occurrence of moderate and severe BCRL compared to the younger age group. With advancing age, there may be natural changes in the lymphatic system that affect its ability to efficiently drain lymph fluid. This reduced lymphatic function can increase the risk of developing lymphedema and potentially lead to more severe symptoms [28]. Furthermore, older individuals are more likely to have multiple comorbid conditions, such as hypertension, diabetes, or cardiovascular diseases [29]. These comorbidities can affect overall health and compromise the lymphatic system’s functionality, making them more susceptible to developing severe lymphedema [30]. Aging is also associated with a decline in skin elasticity and tissue tone. This loss of elasticity can impede the ability of tissues to accommodate fluid accumulation, leading to increased swelling and severity of lymphedema symptoms [31]. Lastly, older individuals generally have a slower healing process and may take longer to recover from surgery. This delayed healing can exacerbate lymphatic dysfunction and contribute to the severity of lymphedema symptoms [32]. The results of conducted research show a wide range of BCRL occurrence frequencies. Differences in study quality, sample size estimation, sampling technique, and research methodology usually form the basis for heterogeneity in meta-analysis of data on incidence or prevalence [33–36]. The relatively low incidence of BCRL in our study can be explained by the specific algorithm of early rehabilitation implemented at the Institute of Oncology of Vojvodina in Sremska Kamenica. The early rehabilitation program begins on the second day after breast cancer surgery, provided there are no surgical or cardiological contraindications [21]. Patients are trained in exercises from the early rehabilitation program with the aim of preventing the development of functional complications through active movement, stretching of the postoperative scar, and activation of the “muscle pump.” Active exercises allow for proper remodeling of the postoperative scar in the axillary region and preservation of functionality in the ipsilateral arm. The kinesiotherapy set consists of 5 to 6 exercises performed daily, 2 to 3 times a day, including rhythmic flexion and extension, as well as circular movements at all levels of the ipsilateral arm, in standing and lying positions with the elevated arm, without fatigue, under the supervision of a physiotherapist during hospitalization. Education during early rehabilitation is intended for patients and family members with the aim of maintaining or restoring functional status and/or maximizing the level of functional independence while minimizing the effects of the disease and its treatment [22]. Numerous published results report the benefits of early rehabilitation interventions precisely during this period [8, 37, 38]. The positive effects are manifested by increased range of motion in the direction of flexion and abduction in the shoulder joint, both in the short term (3 months after surgery) and in the long term (1 year after surgery), without an increased risk of complications such as seroma formation, wound healing difficulties, postoperative pain, and BCRL [37, 38].

This retrospective study provides valuable insights into the occurrence and severity of secondary lymphedema following breast cancer surgery among female patients in Sremska Kamenica, Serbia. The findings highlight the importance of long-term surveillance and support for breast cancer survivors, as lymphedema can manifest several years after the initial treatment. The study provides important baseline data on secondary lymphedema in this population, highlighting the need for ongoing research, advancements in treatment modalities. Early intervention, such as early rehabilitation can help reduce swelling, improve lymphatic function, and alleviate symptoms. The findings underscore the importance of multidisciplinary care involving healthcare professionals who specialize in the management of lymphedema. Ultimately, improving the quality of life for individuals affected by secondary lymphedema remains a crucial goal in the field of oncology.

### Limitations

This study had several limitations. The study was conducted on a relatively small sample size of 150 female patients from a specific region (Novi Sad, Serbia). This may limit the generalizability of the findings to other populations. The study utilized a retrospective cross-sectional design, relying on data collected from medical records and patient charts. This design may introduce biases and limitations associated with retrospective data collection, including missing or incomplete information. The study was conducted in a single center, which may limit the representativeness of the findings. The results may not reflect the experiences and outcomes of breast cancer survivors in other healthcare settings or regions. The diagnosis of lymphedema was based on clinical assessment by healthcare professionals, including physical examination and patient-reported symptoms. While objective measurements were utilized when available, the reliance on clinical assessment alone may introduce subjectivity and potential variability in the diagnosis.

## Conclusion

In our study, the type of surgery, elapsed time since surgery, and the number of removed lymph nodes were not influencing factors for the occurrence of BCRL. However, concerning its severity, a greater number of systemic therapy modalities combined with radiotherapy were associated with a more frequent occurrence of mild and moderate BCRL. Also, the severity of BCRL varied among different age groups, with a higher incidence of moderate and severe lymphedema observed in patients aged 61 years and older.

## Data Availability

Data and materials can be obtained by reaching out to the corresponding author upon request.

## References

[1] Sleigh BC, Manna B. Lymphedema. 2023 Apr 19. In: StatPearls [internet]. Treasure Island (FL): StatPearls publishing; 2023 [internet]. [cited 2023 may 22].

[2] Rockson SG (2018). Lymphedema after breast Cancer treatment. N Engl J Med..

[3] Rockson SG, Keeley V, Kilbreath S, Szuba A, Towers A (2019). Cancer-associated secondary lymphoedema. Nat Rev Dis Primers..

[4] He L, Qu H, Wu Q, Song Y (2020). Lymphedema in survivors of breast cancer. Oncol Lett..

[5] McDuff SGR, Mina AI, Brunelle CL, Salama L, Warren LEG, Abouegylah M (2019). Timing of lymphedema after treatment for breast cancer: when are patients most at risk?. Int J Radiat Oncol Biol Phys..

[6] Asdourian MS, Swaroop MN, Sayegh HE, Brunelle CL, Mina AI, Zheng H (2017). Association between precautionary behaviors and breast cancer-related lymphedema in patients undergoing bilateral surgery. J Clin Oncol..

[7] McEvoy MP, Ravetch E, Patel G, Fox J, Feldman S (2021). Prevention of breast cancer-related lymphedema. Clin Breast Cancer..

[8] DiSipio T, Rye S, Newman B, Hayes S (2013). Incidence of unilateral arm lymphoedema after breast cancer: a systematic review and meta-analysis. Lancet Oncol..

[9] Tsai RJ, Dennis LK, Lynch CF, Snetselaar LG, Zamba GK, Scott-Conner C. The risk of developing arm lymphedema among breast cancer survivors: a meta- analysis of treatment factors. Ann Surg Oncol. (2009) 16(7):1959–72. 10.1245/s10434–009–0452-2. 10.1245/s10434-009-0452-2 19365624

[10] Asdourian MS, Skolny MN, Brunelle C, Seward CE, Salama L, Taghian AG (2016). Precautions for breast cancer-related lymphoedema: risk from air travel, ipsilateral arm blood pressure measurements, skin puncture, extreme temperatures, and cellulitis. Lancet Oncol..

[11] Ferguson CM, Swaroop MN, Horick N, Skolny MN, Miller CL, Jammallo LS (2016). Impact of ipsilateral blood draws, injections, blood pressure measurements, and air travel on the risk of lymphedema for patients treated for breast cancer. J Clin Oncol..

[12] McLaughlin SA, Wright MJ, Morris KT, Sampson MR, Brockway JP, Hurley KE (2008). Prevalence of lymphedema in women with breast cancer 5 years after sentinel lymph node biopsy or axillary dissection: patient perceptions and precautionary behaviors. J Clin Oncol..

[13] Executive C (2016). The diagnosis and treatmen to peripheral lymphedema:2016 consensus document of the international society of lymphology. Lymphol..

[14] Armer JM, Hulett JM, Bernas M, Ostby P, Stewart BR, Cormier JN (2013). Best practice guidelines in assessment, risk reduction, management, and surveillance for post-breast cancer lymphedema. Curr Breast Cancer Rep..

[15] Ostby PL, Armer JM, Dale PS, Van Loo MJ, Wilbanks CL, Stewart BR (2014). Surveillance recommendations in reducing risk of and optimally managing breast cancer-related lymphedema. J Pers Med..

[16] Harrington S, Gilchrist L, Sander A (2014). Breast cancer EDGE task force outcomes: clinical measures of pain. Rehabil Oncol..

[17] Soran A, Ozmen T, McGuire KP, Diego EJ, McAuliffe PF, Bonaventura M (2014). The importance of detection of subclinical lymphedema for the prevention of breast cancer-related clinical lymphedema after axillary lymph node dissection; a prospective observational study. Lymphat Res Biol..

[18] Goss JA, Greene AK (2019). Sensitivity and Specificity of the Stemmer Sign for Lymphedema: A Clinical Lymphoscintigraphic Study. Plast Reconstr Surg Glob Open..

[19] McLaughlin SA, Staley AC, Vicini F, Thiruchelvam P, Hutchison NA, Mendez J (2017). Considerations for clinicians in the diagnosis, prevention, and treatment of breast cancer-related lymphedema: recommendations from a multidisciplinary expert ASBrS panel : part 1: definitions, assessments, education, and future directions. Ann Surg Oncol..

[20] Tomić S, Malenković G, Lalić N, Bojović M, Tomić S. Effects of early rehabilitation treatment on the functional recovery and quality of life in patients three months after breast cancer surgery. Srp Arh Celok Lek. 2020;148(1–2):81–6.

[21] Popović-Petrović S, Tomić S, Nedeljković M, Popović L, Matovina GEarly rehabilitation in patients with breast carcinoma. Vojnosanit Pregl. 2013;70(4):407–10. 10.2298/vsp1304407p 23700947

[22] Yen TW, Fan X, Sparapani R, Laud PW, Walker AP, Nattinger AB (2009). A contemporary, population-based study of lymphedema risk factors in older women with breast cancer. Ann Surg Oncol..

[23] Paskett ED, Naughton MJ, McCoy TP, Case LD, Abbott JM (2007). The epidemiology of arm and hand swelling in premenopausal breast cancer survivors. Cancer Epidemiol Biomarkers Prev..

[24] Meeske KA, Sullivan-Halley J, Smith AW, et al. Risk factors for arm lymphedema following breast cancer diagnosis in black women and white women. Breast Cancer Res Treat. 2008;113(2):383-91. 10.1007/s10549-008-9940-5 18297429

[25] Vicini F, Shah C, Lyden M, Whitworth P (2012). Bioelectrical impedance for detecting and monitoring patients for the development of upper limb lymphedema in the clinic. Clin Breast Cancer..

[26] Engel J, Kerr J, Schlesinger-Raab A, Sauer H, Holzel D (2003). Axilla surgery severely affects quality of life: results of a 5-year prospective study in breast cancer patients. Cancer Res Treat..

[27] Kim HK, Ju YW, Lee JW, Kim KE, Jung J, Kim Y (2021). Association between number of retrieved sentinel lymph nodes and breast cancer-related lymphedema. J Breast Cancer..

[28] Shang T, Liang J, Kapron CM, Liu J (2019). Pathophysiology of aged lymphatic vessels. Aging..

[29] Petrie JR, Guzik TJ, Touyz RM (2018). Diabetes, hypertension, and cardiovascular disease: clinical insights and vascular mechanisms. Can J Cardiol..

[30] Jiang X, Tian W, Nicolls MR, Rockson SG. The lymphatic system in obesity, insulin resistance, and cardiovascular diseases. Front Physiol. 2019:10:1402. 10.3389/fphys.2019.01402. 10.3389/fphys.2019.01402 PMC6868002 31798464

[31] Li K, Zhang Z, Liu NF, Feng SQ, Tong Y, Zhang JF (2017). Efficacy and safety of far infrared radiation in lymphedema treatment: clinical evaluation and laboratory analysis. Lasers Med Sci..

[32] Guo S, DiPietro LA (2010). Factors affecting wound healing. J Dent Res..

[33] Iyigun EZ, Selamoglu D, Alco G, Pilanci KN, Ordu C, Agacayak F (2015). Bioelectrical impedance for detecting and monitoring lymphedema in patients with breast cancer. Preliminary results of the Florence nightingale breast study group. Lymphat Res Biol..

[34] Bundred NJ, Stockton C, Keeley V, Riches K, Ashcroft L, Evans A (2015). Comparison of multi-frequency bioimpedance with perometry for the early detection and intervention of lymphoedema after axillary node clearance for breast cancer. Breast Cancer Res Treat..

[35] Stout Gergich NL, Pfalzer LA, McGarvey C, Springer B, Gerber LH, Soballe P (2008). Preoperative assessment enables the early diagnosis and successsful treatment of lymphedema. Cancer..

[36] Sun F, Skolny MN, Swaroop MN, Rawal B, Catalano PJ, Brunelle CL (2016). The need for preoperative baseline arm measurement to accurately quantify breast cancer-related lymphedema. Breast Cancer Res Treat..

[37] Ammitzbøll G, Kjaer T, Johansen C, Lanng C, Wreford Andersen E, Kroman N (2019). Effect of progressive resistance training on health-related quality of life in the first year after breast cancer surgery-results from a randomized controlled trial. Acta Oncol..

[38] Ayre K, Parker C (2019). Lymphedema after treatment of breast cancer: a comprehensive review. J Unexplored Med Data..

